# Supporting Podiatric Medicine Education Through Tailored Work Allocation Models

**DOI:** 10.1002/jfa2.70139

**Published:** 2026-02-24

**Authors:** A. Coda, A. Fellas, C. Escalona‐Marfil, X. Ruiz‐Tarrazo, X. Ortas, X. Girones, F. Hawke

**Affiliations:** ^1^ School of Health Sciences College of Health, Medicine and Wellbeing—The University of Newcastle Newcastle New South Wales Australia; ^2^ Faculty of Health Sciences at Manresa University of Vic—Central University of Catalonia (UVic‐UCC) Manresa Spain; ^3^ University of Girona Cultura i Educació Research Group Girona Spain; ^4^ Department of Research and Universities Government of Catalonia—Generalitat de Catalunya Barcelona Spain

**Keywords:** clinical education, podiatric medicine, student‐to‐staff ratio, work allocation models, workforce shortage

## Abstract

This commentary explores the necessity of tailored work allocation models in podiatric medicine education to meet the unique demands of clinical programmes. It highlights the importance of maintaining optimal student‐to‐staff ratios, the impact on student development, programme reputation and addressing the national workforce shortage in podiatry.

## 1 Introduction

Academic workload models are frameworks used by universities to allocate and manage the work of academic staff. These models aim to ensure a fair and transparent distribution of tasks, balancing teaching, research, administration/engagement and other responsibilities. These models help universities manage resources effectively and support academic staff in balancing their diverse responsibilities. However, a ‘one‐size‐fits‐all’ approach to work allocation models may not suit all academic programmes, particularly those with significant clinical components, such as Podiatric Medicine programs. While academic workload models are designed to balance academic staff’s responsibilities, the unique demands of clinical programmes require greater flexibility to meet the Australian Health Practitioner Regulation Agency (AHPRA) accreditation standards. Delivering adequate clinical education is essential for preparing graduates who are ready to enter the workforce.

Programmes like Podiatric Medicine provide students with crucial clinical experiences, including placements in rural and public health settings, and provide them opportunities to treat patients with complex medical conditions and lower limb problems [1, 2]. Podiatry graduates are highly sought after due to the depth of clinical training they receive in both the private and public settings [3]. However, without a tailored work allocation that supports the delivery of suitable podiatric educational standards, these programmes may risk compromising both patient outcomes and teaching quality, ultimately affecting student preparedness, employability and the university's reputation. Thanks to the continued institutional support; academics and clinical supervisors are able to uphold teaching standards and maintain positive course evaluation scores, which contribute to performance reviews and may enhance career progression. It is vital that government and educational leaders account for the distinct needs of diverse and time‐intensive clinical programmes like Podiatric Medicine and maintain adequate flexibility in work allocation. This may ensure accreditation requirements are met and helps produce competent, workforce‐ready graduates.

This commentary examines how continued institutional support has been instrumental in maintaining an optimal student‐to‐staff ratio in podiatric programmes, emphasising its positive impact on student development, programme reputation and efforts to address the national workforce shortage in podiatry.

Finally, this commentary advocates for flexible academic workload allocation models that are fit‐for‐purpose for clinically intensive programmes, recognising the distinction between institutional workload models governing continuing academic staff and clinical staff‐to‐student ratios set for patient safety during placements.

## 2 Discussion

### 2.1 Addressing the Need for Flexibility in Work Allocation Models

Government and educational leaders responsible for creating work allocation models should keep considering that a ‘one‐size‐fits‐all’ approach may not be sustainable across all academic programmes. The teaching requirements for degrees such as engineering, accounting, law and podiatry can vary significantly, especially regarding the educational components essential for professional accreditation, where relevant. Each of these fields has unique demands that must be met to ensure students receive the comprehensive education necessary for their future careers.

Work allocation models are typically designed to maximise the university’s resources and ensure an equitable balance between academic staff's responsibilities in teaching, research and engagement. However, the unique demands of certain programmes, especially those with clinical requirements should necessitate greater flexibility in these models. For example, podiatric medicine students are expected to complete significant practical experience during their second, third and fourth years of study, with a focus on meeting the professional capabilities required for graduation. Although the previous standard of 1000 h is no longer a strict requirement, programmes must now ensure and demonstrate that graduating students meet the necessary accreditation standards based on professional capabilities. New programmes are currently being assessed against these updated standards [4]. This practical competency‐based experience is a critical element in producing ‘life‐ready’ graduates who are prepared to transition into the workforce, particularly as new podiatry graduates are allowed to work unsupervised within the full scope of practice [5].

In the last decade, podiatry programmes around Australia have faced possible closure due to issues around financial sustainability, as they tend to have small cohorts of students with clinical‐intensive (and therefore supervision‐intensive) training [6, 7, 8]. The podiatric medicine programme, in particular, not only provides students with extensive clinical experiences, delivered internally but also incorporates external placements, including those in rural and regional areas [9]. Graduates of this programme are in high demand because they are trained to be safe and independent clinicians [2]. Thanks to the tailored work allocation approaches provided by their respective institutions; programmes with significant clinical components have excelled in maintaining and expanding student‐led clinics, even with smaller cohorts than the past. This support has ensured both high‐quality patient outcomes and an enriched student learning experience. By investing in podiatric medicine educators, institutions help ensure students are well prepared for professional practice and uphold the university’s reputation. Furthermore, such support enables academics to maintain strong teaching standards and positive evaluation scores, which are crucial for performance reviews and career progression [10].

As a case illustration, the Podiatric Medicine programme at the University of Newcastle has implemented institutionally endorsed workload principles that formally recognise clinical teaching, placement coordination and engagement activities supporting an 18‐chair student‐led clinic usually operating 4 days per week during semester time. This experience is only presented as a case, not a generalisation, acknowledging variability across the sector. Furthermore, this commentary outlines the following emerging models that influence academic workload planning in clinically intensive programmes:

- simulation‐enhanced curricula to develop clinical skills and patient safety competencies,
- degree apprenticeships combining paid employment with academic study and
- hybrid/remote block delivery (e.g., Australian programmes offering predominantly online learning with intensive on‐campus blocks).

Each model reshapes the distribution and nature of academic responsibilities, such as curriculum design, placement coordination, assessment and quality assurance, while maintaining the essential requirement for appropriate clinical supervision ratios.

Clinical education in Australia is delivered through diverse models. Some programmes operate student‐led university clinics; others rely primarily on block placements in public or community services and simulated learning environments. Typically, academic workload allocation models assign percentages of an ongoing academic's time across teaching, research and service. Clinical staff‐to‐student ratios, by contrast, are operational safety parameters for supervised practice (often met through sessional or casual clinical educators). These concepts should be clearly distinguished: tailored workload models govern the allocation of academic tasks, whereas mandated clinical supervision ratios remain essential for patient safety. In recent years, targeted government funding for podiatry clinical education has been limited, and several programmes have entered teach‐out or consolidation phases. These sector pressures underscore the need to continue implementing institutional strategies that safeguard safe and sustainable clinical teaching.

### 2.2 Staff‐to‐Student Ratio in Podiatric Education and Clinical Competency

Over the past 17 years, the University of Newcastle (Australia) podiatry programme has used a 1:8 staff‐to‐student ratio in student‐led clinics to supervise podiatry students during routine consultations; however, variation may occur between different institutions. As part of daily university clinics, students commonly perform a variety of tasks such as chronic wound assessment, sharp debridement with a scalpel, administration of local anaesthetics, assessment of children's joints, prescription of rehabilitation exercises and corrective foot orthoses. Maintaining an optimal staff–student ratio ensures that students are not overwhelmed while learning to treat challenging podiatric clinical cases.

This structured exposure not only ensures clinical safety and enhances student experience and engagement but also upholds the standard of care that students are expected to demonstrate as part of their clinical course. This ratio allows supervisors to closely monitor and mentor students, ensures that the balance between theoretical knowledge and practical application is maintained and provides opportunities for timely feedback, consolidation and application. This delicate balance is critical in preparing life‐ready graduates and ensures that throughout the degree, students become knowledgeable in treating diverse patient care scenarios.

In specialist podiatry sessions, the ratio is even smaller, typically around 1:5, which is essential due to the more complex nature of these patients and the higher clinical responsibilities involved. Specific patient groups that students encounter under this structure include the following:

- Paediatric patients: Students gain valuable insights into growth, development and age‐appropriate care for children, equipping them to address paediatric‐specific podiatric issues. Additionally, special emphasis is given to history taking and consent, which must be formally obtained by the parent or the guardian.
- Sport injuries: Working with athletes who present both acute and chronic injuries, students develop a deep understanding of the unique biomechanical demands and therapeutic approaches required for this population.
- High‐risk patients: Students are trained to provide care that prevents serious complications of chronic diseases such as diabetes and through this can prevent amputation and premature death.
- Surgical cases: Hands‐on exposure to podiatric surgery and the safe administration of local anaesthetics (LA) gives students the technical experience necessary to perform and assist in surgical interventions, a key component of their training in more advanced podiatric care.

Financial constraints in the education sector might inevitably drive considerations to increase the staff‐to‐student ratio. Such measures, if implemented, could severely undermine the quality of education and the learning environment. A diluted ratio compromises the hands‐on supervision and mentorship that is essential to nurturing competent, confident and safe podiatrists. Additionally, clinical supervisors may be placed under significant pressure to perform their clinical duties if there are too many students treating patients. This could lead to higher chances of making genuine mistakes, misdiagnosing, or simply missing signs of critical complications. If litigation arises, the clinical supervisor's registration could be jeopardised, potentially leading to the suspension of their licence and significantly impacting their future career. Universities may also face liability for damages if their workload model is found to have contributed to inadequate supervision. Finally, maintaining the 1:8 ratio for routine podiatric care represents the minimum requirement to ensure clinical safety, serving as a safeguard for both clinical supervisors and the future of the profession. This argument underscores the need for continued investment in Podiatric Medicine programmes to ensure that graduates are adequately prepared to meet the complex demands of modern healthcare, especially as the profession faces an alarming national shortage of skilled podiatrists.

### 2.3 Protecting Reputational Integrity Among Allied Health Professions

Maintaining a 1:8 student‐to‐staff ratio is necessary also for preserving the reputation of Podiatric Medicine programmes among other allied health professions. With podiatrists now formally recognised by AHPRA as the only allied health professionals along with optometrists authorised to prescribe medications, the importance of rigorous training and supervision cannot be overstated. An optimal podiatric supervision ratio provides the foundation for ensuring the following:

- Adequate clinical support and feedback: Students are able to receive personalised guidance and feedback from experienced staff, fostering a supportive and culturally safe learning environment and enhancing their clinical capabilities. This is particularly meaningful during the early stages of the degree, when students are building confidence and facing a steep learning curve in how to best communicate difficult health‐related matters, explain assessment results and provide tailored education to patients. An optimal ratio ensures students are well equipped with the necessary skills and knowledge, helping them meet the highest standards of care and professional practice.
- Professional credibility: New graduates of the Podiatric Medicine programmes become active members of the multidisciplinary team. Therefore, the educational system has a responsibility to maintain the highest standards of clinical excellence, ensuring that podiatry students are well prepared to contribute effectively to patient care and the broader medical field. The preservation of the 1:8 ratio is, therefore, essential to maintaining the integrity and credibility of podiatric education, ensuring that graduates are well prepared to meet the evolving demands of the profession and earn the trust of their multidisciplinary colleagues within the broader healthcare landscape. Given that new graduate podiatrists are permitted to practice to their full scope without supervision, internship, residency or additional licencing exams, preserving the 1:8 ratio, for routine treatment, is even more crucial.

### 2.4 Addressing National Shortage and Future Demand for Podiatrists

In 2023, podiatry was one of only two allied health professions (along with orthoptics) to be classed in Category 1 Workforce Shortage in New South Wales, Australia. It is therefore critical that University podiatry programmes are strong and adequately funded to avoid a potentially crippling shortfall in primary care in NSW, and across Australia. In response to this pressing need, maintaining a 1:8 student‐to‐staff ratio may convey the following strategic signals that educational institutions are committed to:

- Ensuring quality education: Comprehensive training and support ensure that students are fully prepared to excel in their professional roles.
- Attracting future podiatrists: A dedication to student clinical education and professional development enhances the attractiveness of podiatry as a career, encouraging more individuals to pursue the profession, particularly in specific areas, such as sport podiatry and podiatric surgery that could attract more prospective students.
- Meeting healthcare needs: Graduates of Podiatric Medicine programmes that maintain this ratio will be well equipped to serve the growing clinical demand for podiatric services within NSW Health. This is crucial in ensuring a steady pipeline of well‐trained professionals capable of addressing the national shortage and supporting the sustainability of the profession as demand for podiatric services continues to rise both in the public and the private sector.

### 2.5 Alternatives to One‐Size‐Fits‐All Academic Workload Models

It comes as no surprise that university programmes such as Podiatric Medicine that have smaller student cohorts and clinically intensive coursework are expensive for universities to run. Over the last decade, podiatry and podiatric medicine degree programmes around Australia have faced challenges and potential closure due to financial unsustainability. It is also not surprising that universities would attempt to reduce operating costs by reducing the cost of staffing the programme. On face value, this could be achieved by introducing a new academic workload model. However, as detailed above, the ‘*one‐size‐fits‐all*’ approach might best be described as a ‘*one‐size‐fits‐most*’ approach as it simply does not fit a Podiatric Medicine programme. So, what are universities to do? Given the critical workforce shortage, the crucial role podiatrists play in primary health care and the inability to fill workforce shortages through the federal skilled migration programme (as podiatry as a profession does not exist in the main contributors to Australia's skilled migration programme, i.e. India, the Peoples Republic of China and the Philippines), a strong case could be put to the Australian federal government to increase university funding for Podiatric Medicine programmes [11, 12]. Such funding schemes could be used to fund universities to deliver the programmes in such a way that they meet accreditation requirements and could be used to incentivise people to train and become a podiatrist through a bursary model. Evidence suggests that Australia needs more podiatrists and that podiatrists reduce cost to the health budget by reducing the number of people requiring amputation due to complications from diabetes [13]. The ageing population of Australia [14], with associated increasing prevalence of chronic disease such as diabetes and related complications such as foot ulceration, will drive future demand for podiatrists and necessitate a robust pipeline of skilled podiatrists to meet these healthcare demands [2]. It would be regrettable, and contrary to the national interest, if podiatry programmes were perceived as unviable due to challenges in aligning with a standardised academic workload model.

### 2.6 Limitations

This commentary draws on Australia as illustrative cases, reflecting the contexts in which detailed institutional information was available. Workload allocation is a sensitive area, closely linked to governance, staffing and long‐term programme sustainability, and many institutions do not publicly report such data. By focusing on settings where reliable information could be accessed, we provide evidence‐informed insights while acknowledging that these examples do not capture the full diversity of workload allocation approaches used across Australian universities.

## 3 Conclusion

Thanks to continued government and institutional support; academics and clinical podiatry supervisors have successfully sustained the viability and success of Podiatric Medicine programmes. The evidence highlights the critical importance of maintaining optimal staff‐to‐student ratios, which enhance the educational experience, safeguard patient outcomes and uphold institutional reputations. Furthermore, strategic investment in tailored workload models supports broader efforts to address the national shortage of skilled podiatrists, ensuring that the healthcare system remains robust and responsive to the growing demands of an ageing population. To date, there has been limited targeted government funding specifically for podiatry clinical education in recent years, and several programmes have entered teach‐out or consolidation phases. Had institutions provided local, tailored workload arrangements, programmes would have sustained safe clinical teaching despite sector pressures. It is essential that government and educational leaders continue to support Podiatric Medicine programmes, recognising their vital contribution to public health. By doing so, they will not only advance the professional development of future podiatrists but also strengthen the credibility and standing of the institutions that prepare them.

## Author Contributions

**A. Coda:** conceptualization, methodology, writing – original draft, supervision, writing – review and editing. **A. Fellas:** conceptualization, project administration, writing – review and editing. **C. Escalona‐Marfil:** conceptualization, writing – review and editing. **X. Ruiz‐Tarrazo:** conceptualization, writing – review and editing. **X. Ortas:** conceptualization, writing – review and editing, validation. **X. Girones:** conceptualization, writing – review and editing. **F. Hawke:** conceptualization, methodology, writing – review and editing, supervision.

## Funding

The authors have nothing to report.

## Conflicts of Interest

The authors declare no conflicts of interest.

## Data Availability

The data that support the findings of this study are available from the corresponding author upon reasonable request.
